# Streptococcal SpeB Cleaved PAR-1 Suppresses ERK Phosphorylation and Blunts Thrombin-Induced Platelet Aggregation

**DOI:** 10.1371/journal.pone.0081298

**Published:** 2013-11-22

**Authors:** Miriam Ender, Federica Andreoni, Annelies Sophie Zinkernagel, Reto Andreas Schuepbach

**Affiliations:** 1 Division of Surgical Intensive Care Medicine, University Hospital Zurich, University of Zurich, Zurich, Switzerland; 2 Division of Infectious Diseases and Hospital Epidemiology, University Hospital Zurich, University of Zurich, Zurich, Switzerland; Lerner Research Institute, United States of America

## Abstract

**Background:**

The family of 4 related protease-activated receptors (PAR-1, 2, 3 & 4) expressed by mammalian cells allow to sense for and react to extracellular proteolytic activity. Since major human bacterial pathogens secret a wide array of protease(-s) we investigated whether they interfere with human PAR function.

**Methodology/Principal Findings:**

Supernatants from cultures of major human bacterial pathogens were assayed for the presence of protease(-s) capable to cleave overexpressed human PAR-1, 2, 3 and 4 reporter constructs. Group A streptococcus (GAS) was found to secret a PAR-1-cleaving protease. Experiments involving genetical and pharmacological gain and loss of function identified streptococcal pyrogenic exotoxin B SpeB as the protease responsible. On the host’s side analysis of overexpressed PAR-1 carrying alanine substitutions and deletions showed the amino acid residue leucine_44_ on PAR-1’s extracellular N-terminus to be the only cleavage site. Complementary studies on endogenously expressed PAR-1 using PAR-1 blocking antibodies further supported our conclusion. Through PAR-1 cleavage SpeB efficiently blunted thrombin-induced induction of the ERK-pathway in endothelial cells and prevented platelets aggregation in response to thrombin.

**Conclusions/Significance:**

Our results identify a novel function of the streptococcal virulence factor SpeB. By cleaving human PAR-1 at the N-terminal amino acid residue leucine_44_ SpeB rendered endothelial cells unresponsive to thrombin and prevented human platelets from thrombin-induced aggregation. These results suggest that by blunting PAR-1 signaling, SpeB modulates various innate host responses directed against invasive GAS potentially helping the invasive bacteria to escape. This may allow to tailor additional treatments in the future since upon invasion of the blood stream endothelial cells as well as platelets and mononuclear cells respond to PAR-1 agonists aiming to prevent further bacterial dissemination.

## Introduction

Group A Streptococcus (GAS) is one of the top ten pathogens causing infection-related deaths world-wide and is responsible for around 0.5 million deaths annually [1]. GAS has evolved a variety of virulence factors facilitating efficient host colonization and invasion [2]. 

GAS makes use of the host’s clotting network to increase its virulence. Plasminogen activation and recruitment by GAS was for example found to promote the pathogen’s capability to overcome the host’s barriers and to facilitate blood stream infection [3]. Also the host’s capacity to form fibrin clots is crucial to contain bacterial spread after blood stream invasion [4,5]. On the other hand increased clot formation was shown to boost bacterial dissemination [6]. Recently, studies exploring the bacteria-host interaction from the host’s side showed that a clotting enzyme receptor, the protease activated receptor (PAR)-1, impairs survival in a mouse *pneumococcal* pneumonia model [5] further underlining the interplay between bacteria and the host’s clotting components in the modulation of bacterial virulence. 

PARs consist of a family of 4 highly related G protein-coupled receptors, abundantly expressed on almost all mammalian cells [7]. PARs allow cells to sense for extracellular enzymatic activity [8] through a unique proteolytic receptor activation mechanism. PAR molecules contain hidden activation ligands within their extracellular N-terminus. Proteolytic removal of N-terminal peptides expose neo-amino N-termini that serve as tethered ligands either activating the same receptor molecule [9] or an adjacent PAR molecule [10], thereby initiating transmembrane signaling. Recently PAR-1 was shown to carry several cleavage sites which uncover various signalling-competent tethered ligands causing ligand-specific biological effects [11–13]. 

The impact of PAR-1 activation by mammalian proteases and the resulting effects on systemic inflammation has been extensively studied [14–19]. This receptor was found to have important effects on regulating and maintaining the vascular barrier integrity [18], cytokine secretion [20], apoptosis [11,14] and cell proliferation [21]. However studies on how the initiators of systemic inflammation such as bacterial pathogens impact PAR-1 are scarce [22]. So far it was found that the pathogen *Porphyromonas gingivalis* causing local infections such as periodonitis promotes platelet activation [23] and that *Pseudomonas aeruguinosa* activates PAR-1 and mediates thrombin-like biological effects [24]. However, to our knowledge, major human Gram positive bacterial pathogens responsible for the majority of systemic bacterial infections and consecutive systemic inflammation have not yet been reported to affect PAR-1. 

Herein we studied whether the human bacterial pathogen GAS responsible for up to 0.66 million yearly systemic infections worldwide [1], affects PAR-1. We found that the GAS secreted cysteine protease streptococcal pyrogenic exotoxin B (SpeB) efficiently cleaved PAR-1. We identified its specific cleavage site and studied biological downstream effects. We showed that SpeB attenuated extracellular-signal-regulated kinase (ERK) phosphorylation and rendered PAR-1 unresponsive to thrombin and thereby blunted platelet activation. 

## Results

### Specific cleavage of PAR-1 by group A streptococcal supernatants

**Figure 1:**
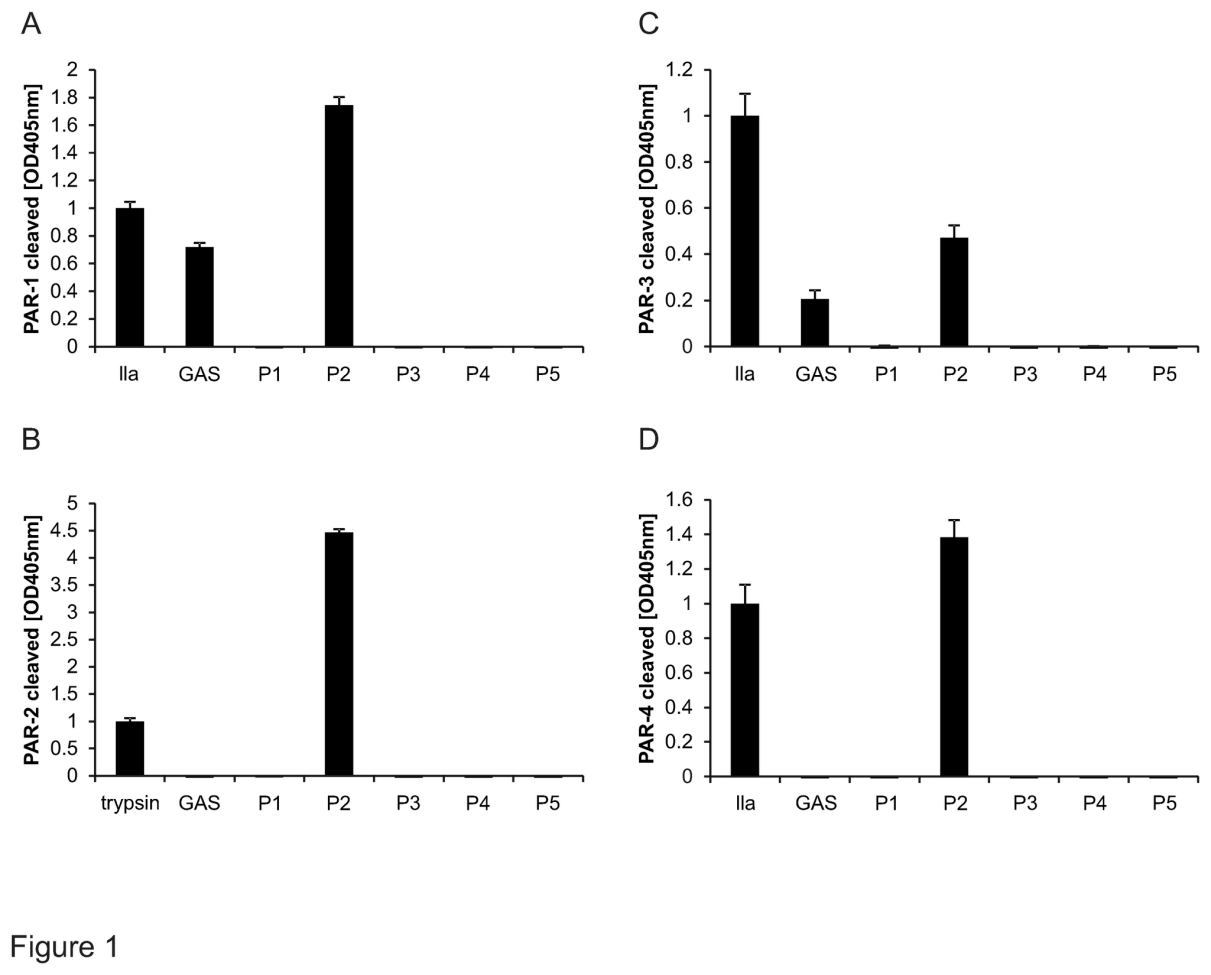
Screening of bacterial supernatants for cleaving PAR reporter constructs. 293T cells transiently over-expressing alkaline phosphatase tagged PAR-1 (A), PAR-2 (B); PAR-3 (C) or PAR-4 (D) were incubated with bacterial supernatants of GAS as well as other pathogenic bacteria P1-P5 (P1 representing *Enterococcus cloacae*, P2 *Pseudomonas aeruginosa*, P3 *Klebsiella pneumonia*, P4 *Burkholderia cepacia* complex, and P5 Methicillin-resistant *Staphylococcus aureus*). Positive controls used for PAR-1, 3 and 4 was thrombin (IIa) and for PAR-2 trypsin. Released alkaline phosphatase activity was quantified. Representative experiment of 3 with N=9, data presented as mean +/- SEM.

In order to test whether GAS secretes proteases capable of cleaving PARs we relied on a cleavage reporter system we had previously used to characterize human serine proteases [11,25]. In brief all four human PARs encoding mRNAs were cloned and the signal sequences were replaced by stop codon-truncated mRNA encoding for secreted alkaline phosphatase (AP). The resulting chimeric PARs carrying a N-terminal AP were successfully expressed in transiently transfected 293T cells and used to screen whether supernatants of relevant human pathogens such as GAS specifically cleaved one of the 4 human PARs. Among the bacterial supernatants screened we identified GAS to efficiently and selectively cleave PAR-1 (Figure 1A) and confirmed earlier studies showing that *P. aeruginosa* cleaves PAR 1, 2, 4 [24,26] and less efficiently PAR-3 (Figure 1A-D). 

### PAR-1 cleavage by streptococcal SpeB

**Figure 2:**
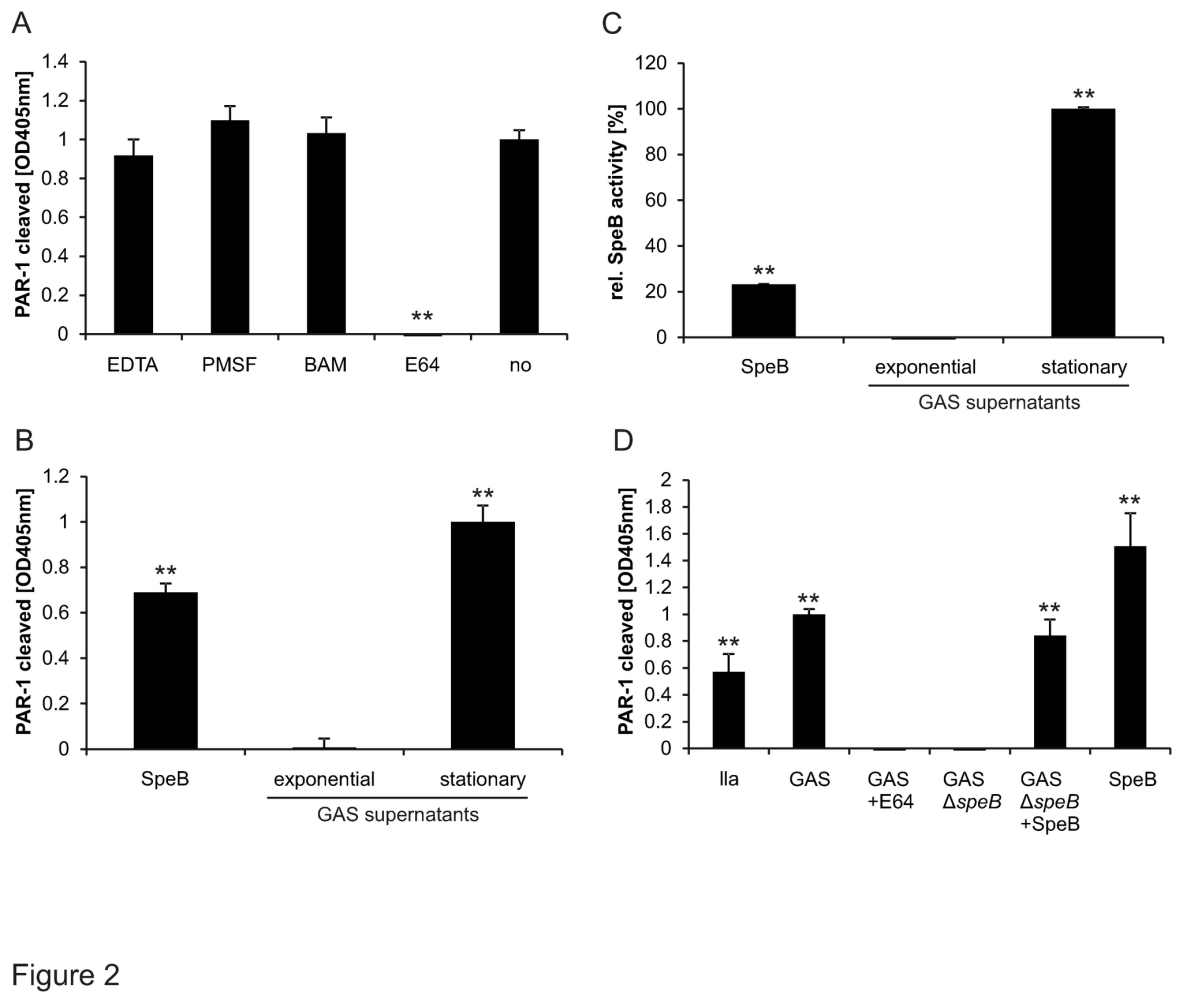
Streptococcal protease responsible for PAR-1 cleavage. (**A**) GAS supernatants pre-incubated with either the metalloprotease inhibitor EDTA, the serine protein inhibitors PMSF and benzamidine (BAM), the cysteine protease inhibitor E64 or buffer were added onto 293T cells transiently over-expressing alkaline phosphatase-tagged PAR-1. Released alkaline phosphatase activity was quantified in the supernatants. (**B**) Cells as described in (A) were incubated with supernatants from exponential and stationary GAS to analyse their efficiency in cleaving AP-PAR-1 reporter constructs. (**C**) SpeB proteolytic activity was analysed by Bz-Pro-Phe-Arg-Nan cleavage in the exponential and stationary cultures GAS samples used in (B). Commercial SpeB (13.3 µg/ml) served as a positive control. (**D**) AP-PAR-1 reporter constructs were incubated with overnight cultures of non pre-treated wild type GAS, GAS preincubated with cysteine protease inhibitor E64 or the isogenic speB deficient GAS (GAS∆*speB*). In addition supernatants from GAS∆*speB* were complemented with commercial SpeB (200nM) and cleavage of AP-PAR-1 reporter construct was carried out. Thrombin (IIa, 1nM) served as positive control. Experiments were repeated at least 3 times with N=9, data presented as mean +/- SEM, *P<0.05, **P<0.01.

To identify the streptococcal protease responsible for PAR-1 cleavage we took a stepwise approach. We first used specific protease inhibitors to identify the protease family and found that only the cysteine protease inhibitor E64 blunted PAR-1 cleavage (Figure 2A) consistent with a streptococcal cysteine protease cleaving PAR-1. GAS protease expression is modified according to the growth phase [27]. To narrow down which streptococcal cysteine protease cleaved PAR-1 we thus compared supernatants from exponential and stationary growth phase GAS cultures. We found that the protease was expressed during the stationary growth phase (Figure 2B). Late growth phase enzyme expression together with a zymography (not shown) showing strong proteolytic activity under reducing conditions at around 42 kDa suggested the streptococcal pyrogenic exotoxin B (SpeB) to be the causal protease. Consistent with our hypothesis we detected functional SpeB in supernatants from stationary phase cultures (Figure 2C) using the chromogenic cysteine protease substrate Bz-Pro-Phe-Arg-Nan [28]. To test whether SpeB is required and sufficient for PAR-1 cleavage we analysed supernatants from GAS expressing SpeB (GAS wildtype) or the isogenic SpeB-deficient GAS (GAS∆*speB*) in the absence and presence of exogenously added commercial SpeB. PAR-1 was found to be always cleaved when functional SpeB was present consistent with SpeB being responsible for PAR-1 cleavage (Figure 2D). 

### PAR-1 cleavage restricted to SpeB-expressing clinical isolates

**Figure 3:**
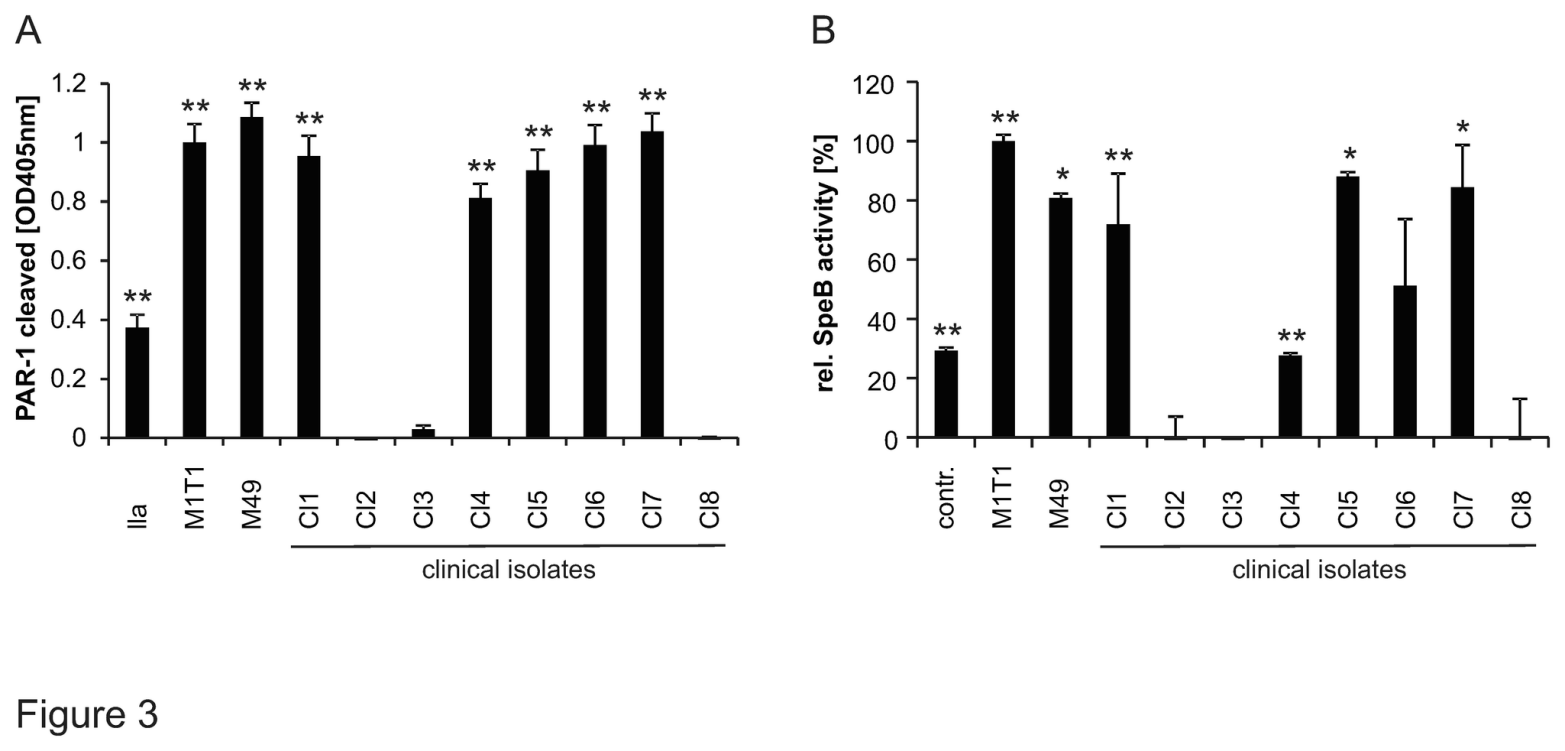
GAS strains cleaving PAR-1 expressed functional SpeB. (**A**) 293T cells transiently expressing alkaline phosphatase tagged PAR-1 were incubated with overnight supernatants of well characterized clinical GAS isolates M1T1, M49 and new clinical GAS isolates (Cl1-8). Then AP-PAR-1 cleavage was assessed. Thrombin (IIa; 1nM) served as positive control. (**B**) Samples from (A) were analysed for proteolytic SpeB activity by evaluating Bz-Pro-Phe-Arg-Nan cleavage. Experiments were repeated at least 3 times with N=9, data presented as mean+/- SEM.

In up to 40% of invasive GAS, the expression of SpeB [3] is strongly reduced due to either point mutation(-s) or deletions within the main GAS two component regulatory system CovR/S or mutations within ropB (also known as Rgg) [29]. Therefore we tested the association between SpeB expression and PAR-1 cleavage in clinical isolates. In addition to the well characterized invasive GAS M1T1 and M49 strains we further tested clinical GAS isolated from patients suffering from invasive GAS infections (Table S1). We tested whether all the GAS strains expressed SpeB and whether they cleaved PAR-1. Among the clinical isolates tested a large proportion (62.5 %; not including the reference strains) cleaved PAR-1 (Figure 3A). All strains that cleaved PAR-1 also secreted functional SpeB (Figure 3B). 

### Cleavage efficiency of SpeB

**Figure 4:**
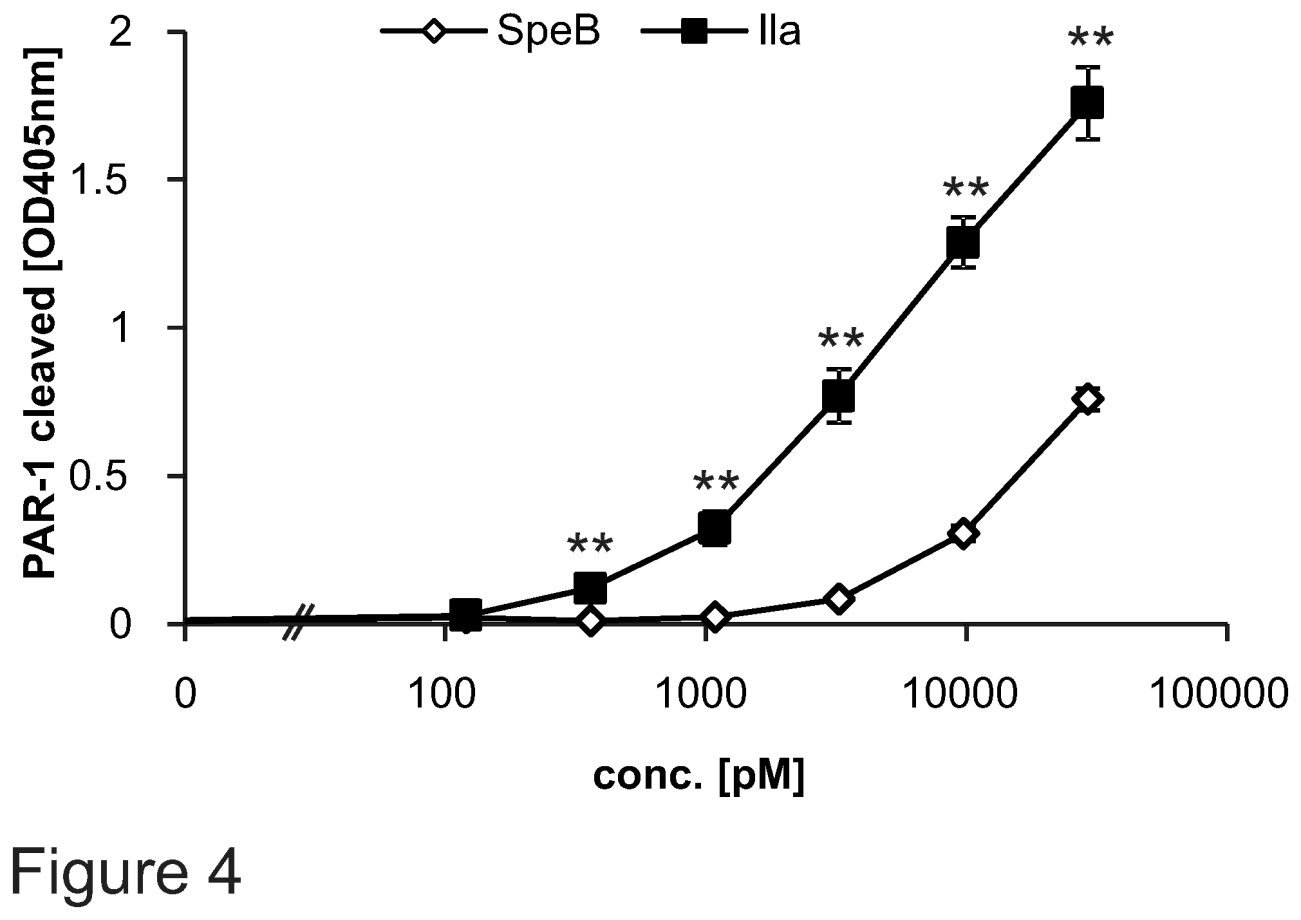
AP-PAR-1 cleavage efficiency by SpeB and thrombin. A large concentration range (starting at 10pM up to 29nM) of commercial SpeB and thrombin (IIa) was analysed for AP-PAR-1 cleavage efficiency. Experiments were repeated at least 3 times with N=9 per point, data presented as mean+/- SEM, *P<0.05, **P<0.01.

We next addressed how efficiently SpeB cleaved PAR-1. Since protein-protein interactions, such as the direct binding of thrombin to PAR-1 [30] and the availability of co-receptors might influence PAR-1 cleavage, we expanded our studies and used cell surface expressed AP-PAR-1. We compared SpeB and thrombin PAR-1 cleavage efficiency and found the latter to be around 9 times more efficient (Figure 4). 

### Cleavage of endogenously expressed PAR-1 by SpeB

**Figure 5:**
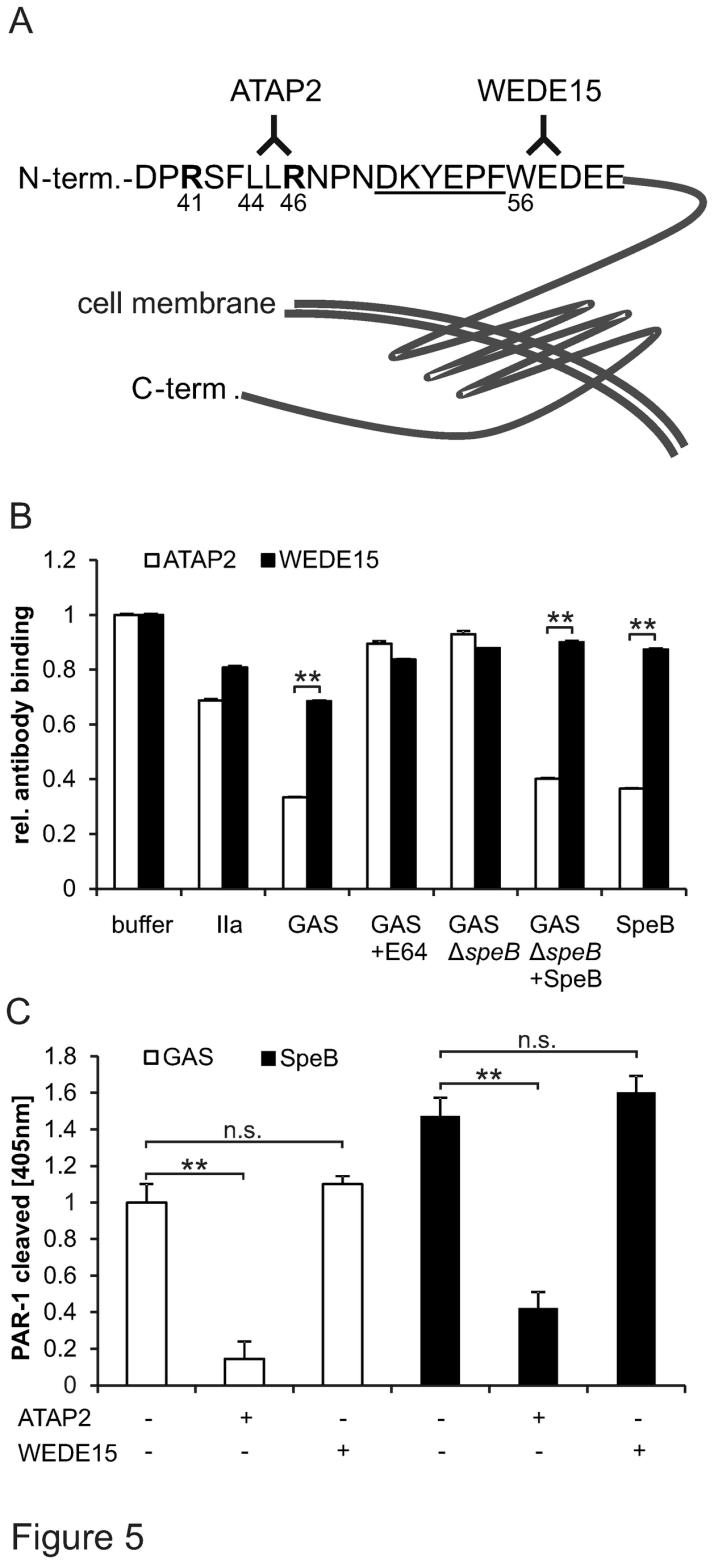
SpeB cleaved endogenous PAR-1 at the ATAP2 epitope and ATAP2 blocked cleavage of overexpressed PAR-1. (**A**) Scheme of PAR-1’s extracellular N-terminus. In bold are given the thrombin and activated protein C cleavage (and activation) sites shown at arginine_41_ and arginine_46_, respectively. Underscores mark the hirudin binding site, the domain directly binding thrombin’s exosite I. Further monoclonal anti-PAR-1 ATAP2 and WEDE15 epitopes are provided. (**B**) Following incubation of endothelial EA.hy926 cells with given agonists, supernatants from GAS or SpeB deficient GAS in the absence or together with protease inhibitor E64 or SpeB cells were fixed with PFA and epitopes of anti-PAR-1 ATAP2 (white bars) and WEDE15 (black bars) were quantified by cell surface ELISA. (**C**) Following incubation with either anti-PAR-1 ATAP2 or WEDE15, 293T cells transiently over-expressing AP-PAR-1 were assessed for PAR-1 cleavage by GAS supernatant and commercial SpeB. Experiments were repeated at least 3 times with N=9 per point, data presented as mean+/- SEM, **P<0.01.

Cleavage of PAR-1’s N-terminus either renders the receptor dysfunctional [31] or induces transmembrane signaling dependent on the cleavage site. Cleavage can in fact occur at several sites and uncover various neo-N-terminal tethered ligands that mediate a variety of divergent biological effects [11–13]. We thus investigated whether SpeB cleaved at a single specific amino residue and its exact cleavage location. We first used monoclonal anti-PAR-1 antibodies and tested whether streptococcal-derived or commercial SpeB removed the anti-PAR-1 epitope from endogenously endothelial cell surface-expressed PAR-1. Anti-PAR-1 ATAP2 binds to the thrombin-generated tethered ligand whereas WEDE15 binds C-terminal of the hirudin-like domain (Figure 5A). We used ATAP2 and WEDE15 to test by cell surface ELISA [32] whether SpeB can remove the ATAP2 or WEDE15 epitope from the N-terminus of endogenously EA.hy926 endothelial cell expressed PAR-1. Both streptococcal*-*derived and commercial SpeB removed ATAP2 epitopes consistent with cleavage within or C-terminal of the ATAP2 epitope [11,32]. In contrast binding of WEDE15 was only weakly affected (Figure 5B). Taken together these data are consistent with SpeB cleaving between R_46_ and W_56_. As a complementary approach we used the same antibodies in our cleavage reporting system and tested whether the presence of either antibody interfered with SpeB cleavage. ATAP2 but not WEDE15 prevented cleavage of both streptococcal-derived and commercial SpeB providing complementary evidence that SpeB cleaved in proximity of the ATAP2 epitope (Figure 5C). In addition and most importantly, cleavage blocking also ruled out that SpeB cleaved AP somewhere between the protease domain and the linkage to PAR-1. 

### Specific cleavage at L_44-45_ by SpeB

To further corroborate our data and to generate additional lines of evidence we constructed a chimeric AP-PAR-1 molecule with a FLAG-tag at the fusion site homologous to the one published previously [33]. Thrombin and streptococcal-derived protease removed the FLAG-tag together with AP whereas supernatants from *speB* deficient GAS did not. This excluded significant cleavage of the reporter construct within the linker region or the AP molecule (Figure S1). 

**Figure 6:**
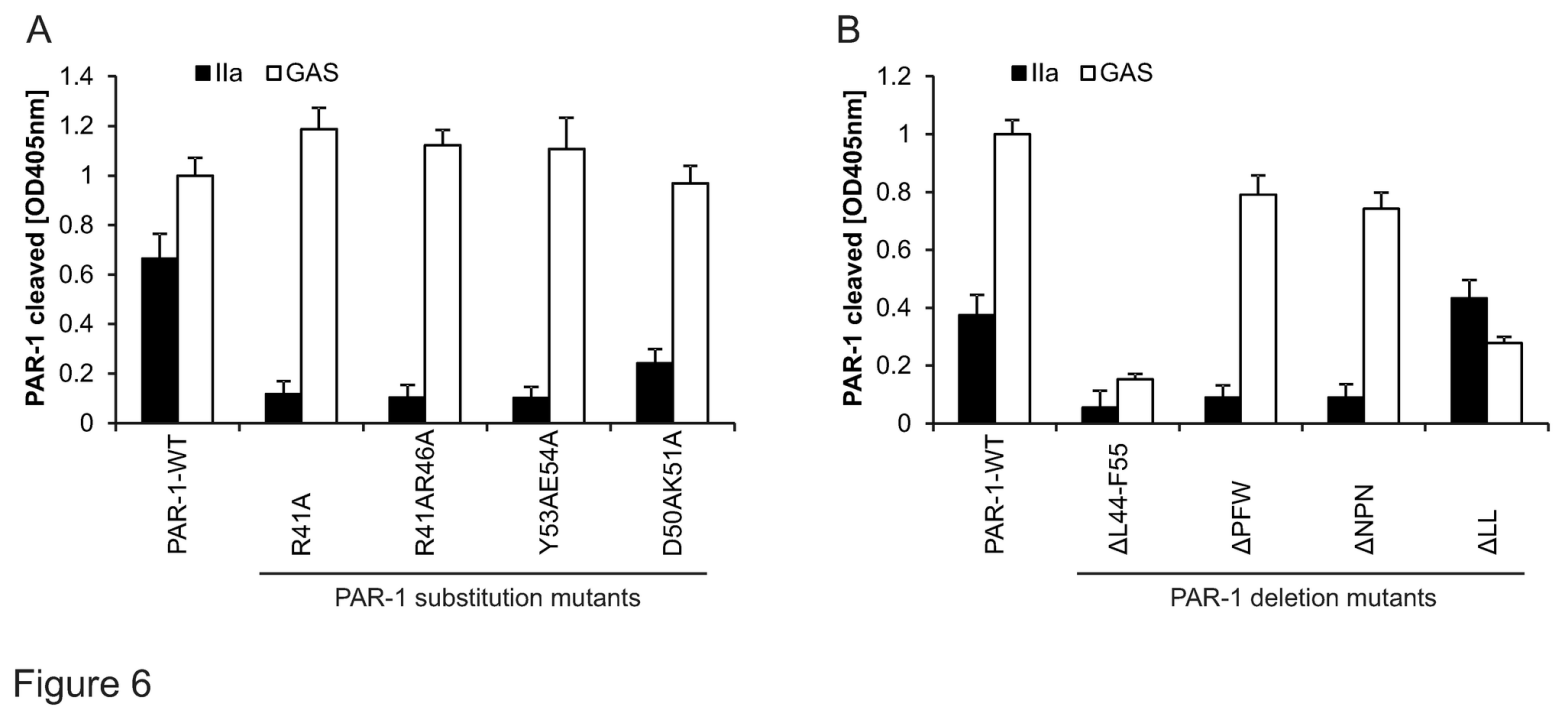
SpeB specifically cleaved endogenous PAR-1 at leucine_44/45_. GAS supernatant and thrombin (IIa; 1nM) were analysed for their ability to cleave (**A**) variants of AP-PAR-1 with alanine substitutions at indicated positions and (**B**) mutants of AP-PAR-1 with deletions at indicated positions. Experiments were repeated at least 3 times with N=9 per point, data presented as mean+/- SEM.

In order to map the exact cleavage site more precisely we constructed numerous variants of AP-PAR-1. None of the mutants known to prevent thrombin-mediated cleavage of PAR-1 resisted SpeB (Figure 6A). However the AP-PAR-1 variant carrying a long deletion between L_44_ and W_56_ was resistant to cleavage consistent with our previous conclusions (Figure 5). Shorter deletions narrowed the cleavage site position to leucine residues 44 and 45 consistent with our data, showing that ATAP2 (epitope L_44_ to R_46_) blocked cleavage. 

### Reduced base line ERK phosphorylation and blunted thrombin responses following SpeB

**Figure 7:**
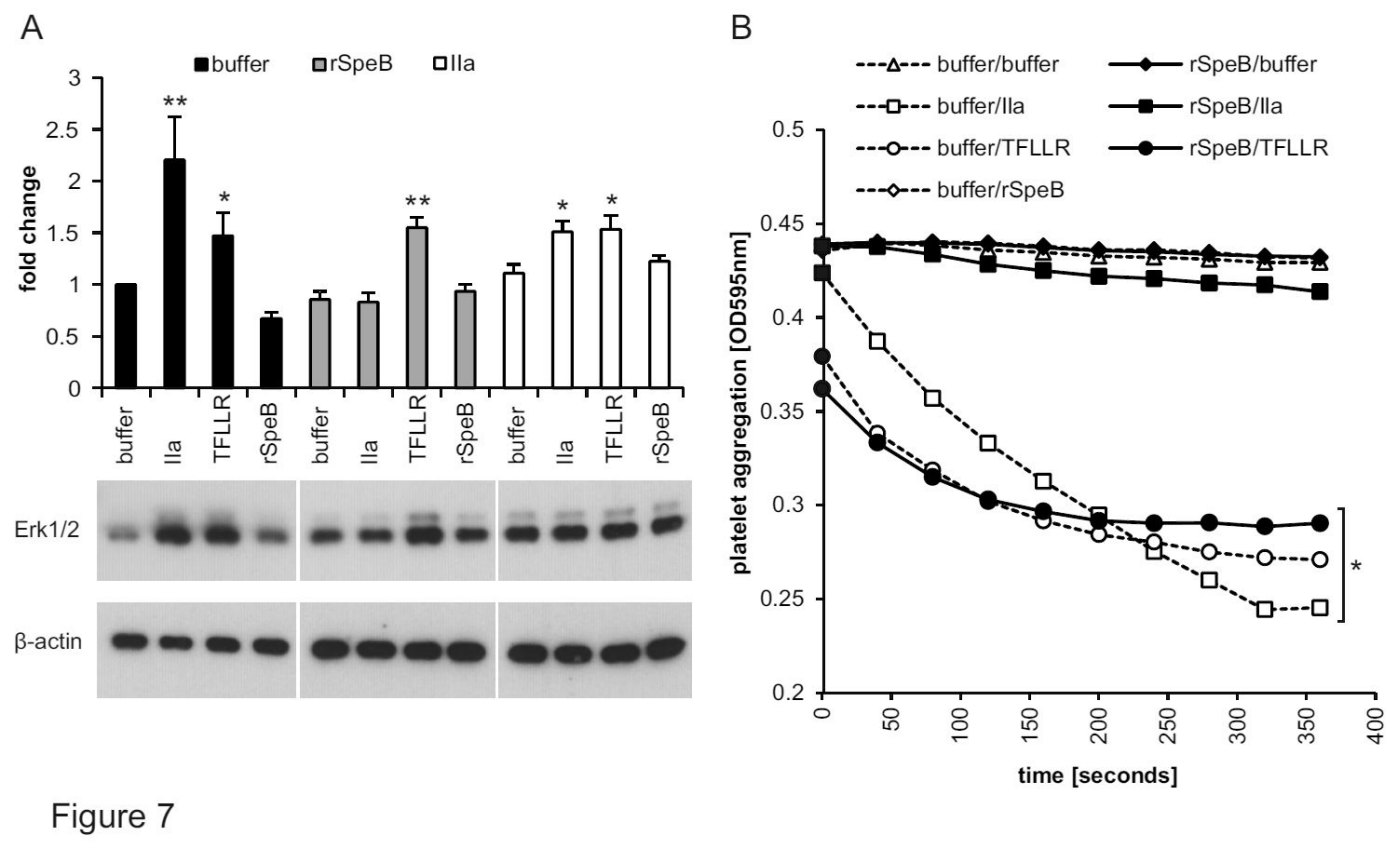
SpeB-cleaved PAR-1 silenced ERK1/2 phosphorylation and blunted thrombin-mediated platelet aggregation. (**A**) Following pre-incubation with buffer alone (open bars), commercial SpeB (grey) or thrombin (black) EA.hy926 endothelial cells were treated with given agonists and assessed by Western blot. β-actin served as loading control. The upper part of the panel provides quantitative analysis of 4 pooled experiments; one-way-ANOVA yielded *P*<0.001 overall, *P*<0.004 for buffer, *P*<0.001 for speB and *P*<0.021 for IIa pre-incubated subgroups. Within each subgroup samples were compared to the corresponding buffer control (Dunnet’s posthoc comparison) *P<0.05, **P<0.01. The lower part of the panel shows a representative blot with visualized bands for phosphorylated ERK and β-actin. (**B**) Washed human platelets were pre-incubated 15 min with buffer alone (open symbols) or commercial SpeB (closed symbols) before addition of indicated agonists and quantification of aggregation. Representative graph of 3 experiments with N=9, data presented as mean+/- SEM, *P<0.05, **P<0.01.

Cleavage at L_44/45_ had been previously described for the human protease cathepsin G [34] without elucidating the biological consequences. We first analysed whether SpeB affects extracellular-signal-regulated kinases (ERK1/2) in EA.hy926 endothelial cells via PAR-1. The prototypical physiological PAR-1 agonist thrombin as well as a synthetic soluble peptide TFLLR which is homologous to the thrombin-generated tethered ligand, induced ERK1/2 phosphorylation whereas SpeB did not (Figure 7A). In cells that had been pre-incubated with commercial SpeB the base line activity of ERK1/2 was even slightly decreased as compared to only buffer treated cells and the thrombin response was blunted. In contrast commercial SpeB pre-incubation did not affect the response to TFLLR consistent with (1) no toxic effects of commercial SpeB and (2) unaffected functionality of PAR-1’s receptor domain. Pre-stimulation with thrombin as a further control resulted in an increased base line activity of ERK1/2 but maintained inducibility by thrombin and TFLLR consistent with only minor desensitization of PAR-1 by the agonist concentrations used.

### Lack of thrombin induced platelet aggregation following SpeB incubation

PAR-1 signaling is crucial for platelet activation. We studied aggregation of washed platelets by turbidimetric analysis to analyze biological effects of SpeB on PAR-1 in platelets. First, aggregation in native platelets (without pre-treatment) was analyzed following exposure to buffer control or thrombin or the soluble PAR-1 agonist TFLLR or commercial SpeB (Figure 7B). In accordance with our previous data showing that SpeB did not induce ERK1/2 (Figure 7A) commercial SpeB also did not induce platelet aggregation, whereas thrombin and TFLLR induced aggregation. We then tested whether pre-treatment with commercial SpeB could influence platelet response to PAR-1 agonists. Consistent with our ERK1/2 phosphorylation data, pre-incubation with commercial SpeB blunted effects of thrombin whereas platelets fully responded to TFLLR consistent with commercial SpeB rendering the tethered ligand of PAR-1 dysfunctional but not otherwise affecting platelet function. 

## Discussion

We showed that the cysteine protease SpeB, a major GAS virulence factor, cleaved PAR-1. Our results indicate that SpeB specifically cleaved human PAR-1, but neither human PAR-2 nor 4. Human PAR-3 was cleaved very inefficiently. We provide 3 lines of evidence that PAR-1 was cleaved at the single amino acid residue leucine_44_, a cleavage site previously reported to be specific for the human protease cathepsin G [34]. We further found that PAR-1 cleaved at leucin_44_ rendered human endothelial cells and platelets unresponsive to their physiological activator thrombin. 

The PAR-1 cleaving protease present in GAS supernatants was specifically expressed in stationary phase cultures. Experiments involving inhibitors classified this PAR-1 cleaving protease to the cysteine protease family. Using a modified ‘molecular’ Koch’s postulate with bacteria engineered to either express SpeB or not together with complementation experiments adding exogenous commercial SpeB alone or together with GAS supernatant from *speB*-deficient GAS identified SpeB as the protease directly cleaving PAR-1. On the host’s side our studies using PAR-1 overexpressing cells were further confirmed by studies with endogenously PAR-1 expressing endothelial cells. Cell surface ELISA techniques were used to corroborate that SpeB-PAR-1 cleavage was neither an artefact due to overexpressing PAR-1 nor due to specific characteristics of our PAR-1 cleavage reporter construct.

PAR-1 has been recognized to be a ‘Janus like receptor’ given its ability to mediate divergent biological effects dependent on what tethered activation ligand was uncovered through a N-terminal cleavage event [11,13]. Herein we provide complementary evidence that SpeB cleaves PAR-1 at a novel cleavage site (leucine_44_). Overexpressed PAR-1 carrying deletions and endogenous PAR-1 in conjunction with cleavage using blocking anti-PAR-1 antibodies were used. To our knowledge it has so far not been studied whether the tethered ligand of L_44_ cleaved PAR-1 activates either its own receptor body (in cis) or an adjacent PAR 1, 2, 3 or 4 molecule in trans albeit the fact that the site had already been identified for human protease cathepsin G [34]. Multiple active states of PAR1 have been described which depend on whether the receptor was cleaved at R41 or R46 [11,13] resulting in PAR1 activation. Either cleavage site generates a specific tethered ligand that is also specific for biological downstream effects. We now identified an additional novel L_44_ cleaved tethered ligand generated by SpeB. It remains to be clarified whether it induces a novel active state of PAR1 which would resemble the R46 cleaved PAR1 or whether the ligand is shed and thus non-functional. Our data involving studies in a human endothelial cell line and human platelets suggest SpeB to generate an ineffective tethered ligand. Whether such activation ligand destroying (or shedding) effects also blunt PAR-1’s anti-apoptotic effects and whether such shedding might help to explain pro-apoptotic effects of SpeB in human monocyte and lymphocyte like cell lines remains to be clarified [35,36].

SpeB has been shown to be an important GAS virulence factor. On the one hand, SpeB is recognized as a major virulence factor in both *in vitro* and *in vivo* experiments due to its capacity to degrade host matrix protein [37], epithelial junctions [38], immunoglobulin [39–41] and complement [42,43]. On the other hand, diminished SpeB expression caused by mutations within the CovR/S two component system has been linked to invasive GAS strains [3]. Benefits of abolished SpeB expression were so far explained by reduced SpeB-mediated degradation of other GAS virulence factors such as the DNase Sda1. Our observation that SpeB blunts platelet activation adds an additional dimension to SpeB pathogenicity. Reduced SpeB expression is beneficial for blood stream invasion [3] suggesting negative selection of SpeB-expressing GAS in this compartment. One is thus tempted to speculate that the lack of platelet activation by SpeB-expressing GAS within the blood stream might reduce their virulence. GAS is always coated by fibrinogen [44], a plausible mechanism could thus be that SpeB-deficient GAS is preferentially bound to activated platelets promoting micro-thrombosis resulting in tissue invasion.

In summary cleavage of PAR-1 is a novel function of streptococcal SpeB. Our results indicate that among the 4 mammalian expressed PARs (1 to 4) SpeB is specific for PAR-1 and that cleavage occurred at the single amino acid residue leucine_44_. SpeB rendered PAR-1 irresponsive for thrombin in endothelial cells and most importantly rendered platelets unresponsive to their physiological activator thrombin. Further studies will be required to explore the importance of SpeB-cleaved PAR-1 in GAS virulence. 

## Materials and Methods

### Reagents

Clotting proteases were purchased from Haematologic Technologies (Essex Junction, VT, USA), with the exception of Trypsin (Gibco; Life technologies^TM^ Europe, Zug, CH). Commercial SpeB was purchased from Toxin Technology Inc. (Sarasota, FL, USA). PAR-1 activating peptides corresponding to the N-terminus of R41 cleaved PAR-1 (TFLLRNPN) were custom made (Antagene; Sunnyvale, CA, USA) and used at a final concentration of 20µM. Protease inhibitors EDTA, PMSF, benzamidin and E64 were from Sigma-Aldrich Chemie GmbH (Buchs, CH). Monoclonal anti-PAR-1 ATAP2 and WEDE15 were applied as described previously [11,25,32] and used at 1μg/mL for analytic assays and at 25μg/mL for 10 min in cleavage blocking studies. Western blots detecting phosphorylated ERK1/2 were done with the antibodies p44/42 MAPK (#9101, Cell signalling, Inc.; Cambridge, GB) and goat anti-rabbit HRP (#7074, Cell signalling, Inc.; Cambridge, GB) used as described [32], anti-β-actin antibody (Sigma-Aldrich Chemie GmbH; Buchs, CH) and goat anti-mouse HRP (Life technologies Europe; Zug, Switzerland) for β-actin detection and antibodies anti-FLAG M2 (Sigma-Aldrich Chemie GmbH; Buchs, CH) and goat anti-mouse HRP for detecting FLAG-tagged AP-PAR-1. 

### Bacterial strains

The GAS strain 5448, a well-characterized M1T1 clinical isolate from a patient with necrotizing fasciitis and toxic shock syndrome [45] was used as well as clinical isolates from the University Hospital Zurich, Division of Infectious Diseases and Hospital Epidemiology (Table S1). The GAS strain M1T1 lacking the *speB* gene (GASM1T1 Δ*speB*) [46] was used as a control for loss of *speB* function. All GAS strains were grown in Todd Hewitt Broth (BD; Sparks, MD, USA) supplemented with 0.5% yeast extract (THY) at 37°C under static conditions. Other pathogenic bacteria such as *Pseudomonas aeruginosa* were grown in Luria Bertani Broth (BD; Sparks, MD, USA) at 37°C with shaking. Culture supernatants were harvested by centrifugation (4000 rpm, 10 min), filter-sterilized through a 0.22 µm filter (Merck Millipore Ltd.; Cork, IRL), immediately quick frozen in liquid nitrogen and stored at -20°C. Purified SpeB was obtained from stationary phase grown GAS supernatants as described previously [47] and used to compare enzyme activity to commercial SpeB (Figure S2). 

### GAS *emm* typing

*emm* typing was performed by colony PCR using 5’-tattcgcttagaaaattaa-3’ (forward primer) and 5’-gcaagttcttcagcttgttt-3’ (reverse primer) in conjunction with taq polymerase (Sigma-Aldrich). PCR products were sequenced and compared to the CDC data base (http://www.cdc.gov/ncidod/biotech/strep/m-proteingene_typing.htm)

### Cell culture and transfection

EA.hy926 cells [48] and 293T cells were obtained from the American Type Culture Collection (ATCC^®^, CRL-11268^™^) were cultivated and propagated as described previously [32]. For transient over-expression lipofection was performed using expression plasmids (pcDNA3.1/Zeo+ and pcDNA3.1/Hygro+; Life technologies Europe; Zug, Switzerland) and Lipofectamine 2000 (Life technologies Europe; Zug, Switzerland) according to the manufacture’s instruction. Constructs for tagged and non tagged PAR’s were made as described [11,25] and the sequences are provided (Table S2 and Table S3). All mutations and deletions were obtained by site directed mutagenesis using the Phusion® Site-Directed Mutagenesis Kit (NEB; Ipswich, MA, USA). All constructs were verified by sequencing. 

### PAR-1 cleavage reporter assay

293T cells transiently expressing alkaline phosphatase (AP)-tagged PAR cleavage reporter constructs (sequences provided in Table S2 and Table S3), quantification of PAR cleavage and verification of appropriate reporter construct expression was done as reported [11,25]. In brief, cells were washed twice and incubated with pre-warmed agonists (37°C). In all experiments, except from those shown in Figure 1, the agonists were supplemented with DTT (final conc. 1mM; Fermentas/Thermo Scientific; Rockford, IL, USA). Following the agonist incubation of 20 min, supernatants from PAR reporter constructs expressing 293T cells were removed, separated from cell debris by passing them through a cellulose ester membrane (pore size, 0.45 µm; Millipore, Bedford, MA, USA). AP activity was quantified by incubation with the colorimetric substrate p-nitrophenyl phosphate (1-Step PNPP; Thermo Scientific, Rockford, IL, USA) for around 10 min before endpoint measurements with the Labsystems Multiskan MCC/340 plate reader (Thermo Scientific, Rockford, IL, USA).

In all experiments buffer controls were subtracted and used as base line. 

### Cell surface immunoassays and Western blotting

Endothelial EA.hy926 cell surface PAR-1 was quantified by cell surface enzyme-linked immunosorbent assay as described previously[11,25,32]. In brief following agonist incubation cells were PFA (2%) fixed, probed with detection antibody, followed by incubation with HRP-labelled goat-anti mouse antibody and colorimetric quantification using the HRP substrate 3,3',5,5'-Tetramethylbenzidine (Thermo Scientific, Rockford, IL, USA). 

Western blotting was performed as described [32] and antibodies used are provided within the reagent section. In brief proteins were extracted using 2x sample buffer at 80°C, after sonication samples were then separated by SDS-PAGE under reducing conditions, transferred to nitrocellulose (Thermo Scientific, Rockford, IL, USA), blocked, and probed with detection antibody at 1μg/mL followed by HRP-coupled secondary antibody and visualization using the SuperSignal West Femto detection system (Thermo Scientific, Rockford, IL, USA). Optical density of both immunoreactive bands of protein of interest and β-actin for loading control was assessed using the Alpha Innotech FluorChemQ system and the Alpha view software version 2.0.1.1 (Alpha Innotech/Protein simple, Santa Clara, CA, USA).

### Quantification of SpeB enzyme activity

SpeB was quantified in accordance to [28]. Twenty µl of overnight supernatants were mixed with 110 µl of PBS and 10 µl of DTT (final concentration 1mM). After incubation (37°C, 30 min) 20 µl of 2.6mM Bz-Pro-Phe-Arg-Nan (Sigma-Aldrich Chemie GmbH; Buchs, CH) were added and the increase in absorbance at 405 nm was quantified by a kinetic plate reader (BioTek Synergy HT, BioTek Instruments GmbH; Luzern, Switzerland). The maximal slope of the curve was calculated and used to quantify SpeB activity. Commercial SpeB protein (13.3 µg/ml, ToxinTechnology, USA) and THY were used as positive and negative controls, respectively.

### Analysis of soluble N-terminal PAR-1 peptide by mass spectrometry

A soluble PAR-1 peptide covering amino acid 37-64 with an N-terminal biotin-tag and a C-terminal His-tag (ProteoGenix; Schiltigheim, F) was bound to Dynabeads M-280 Streptavidin (Life technologies Europe; Zug, CH) as described in the manufacturer’s instruction. After washing off unbound peptide, beads were incubated with 20 µl GAS M1T1 supernatant or 400 pmol commercial SpeB supplemented with 1mM DTT for 30 min at 37°C. Cleaved (released) C-terminal peptide fragment was then captured with His-Tag Isolation & Pulldown dynabeads (Life technologies Europe; Zug, CH) and eluted as described in the manual followed by analysis by MALDI/MS/MS (Functional Genomic Centre University/ETH Zürich, Switzerland).

### Platelet aggregation assay

Blood was drawn according to the protocol 2010-0126/0 which was approved by the Institutional Review Board of the University of Zurich, Zurich Switzerland, and after written informed consent was obtained from all participants. Citrate blood was supplemented with 7.4 ng/mL Prostine VR (Pfizer AG; Zurich, CH), centrifuged 15 min at 135×*g*; platelet-rich plasma was removed and layered on Histopaque1119 (Sigma-Aldrich Chemie GmbH; Buchs, CH) and 20% human albumin (CSL Behring, Bern, CH). Platelets were further purified by centrifugation (10 min at 100×*g*) followed by a second centrifugation step (35 min 800×*g*). The intermediate platelet layer was then diluted with modified Hepes-tyrods buffer (1:1; 134mM NaCl, 12mM NaHCO_3_, 5mM glucose, 10mM Hepes, 0.34mM NaH_2_PO_4_,pH7.4) and supplemented with 5mM CaCl_2_, 1mM MgCl_2_. Platelets were pre-incubated with Hepes-tyrods buffer or commercial SpeB (endconc. 200nM) in a FCS (Life technologies Europe; Zug, CH) coated 96 well plate (Nunc, immune plates polysorb C96; Thermo Scientific; Rockford, IL, USA) for 15 min at 37°C with shaking. Aggregation was then analysed immediately after addition of agonists by kinetic determination of the absorbance at 595nm while keeping the plate at 37°C shaking (modified from Armstrong PCJ 2009 [49]).

### Statistics

Data analysis and presentation was performed using NCSS, SPSS software packages. A two-sample, two-tailed homoscedastic t-test was used to calculate the indicated *P*-values. Where indicated one-way-ANOVA with Dunnett’s post hoc comparison to the control sample was used. In PAR-cleavage reporter, ELISA and colorimetric assays buffer control samples were subtracted as background *P*-values for platelet aggregation over time was estimated using log rank.

## Supporting Information

Figure S1 **No cleavage of AP-PAR-1 construct outside PAR-1’s N-terminus.** 293T cells transiently expressing alkaline phosphatase and FLAG-tagged PAR-1 were incubated with bacterial supernatants, thrombin (IIa; 1nM) or buffer. Supernatants and cells were then separately analysed by Western blot for N-terminal PAR-1 cleavage. Representative experiment out of 3.
(TIF)

Figure S2 **SpeB column purified from GAS supernatants and commercial SpeB cleaved PAR-1 with comparable efficiency.** 293T cells transiently expressing alkaline phosphatase-tagged PAR-1 were incubated with indicated amounts of column purified (pSpeB) and commercial SpeB and cleavage of PAR-1 reporter constructs was quantified. Thrombin (IIa; 1nM) served as a positive control.
(TIF)

Table S1 **Characteristics of GAS clinical isolates.**
(TIFF)

Table S2 **Amino acid sequences of PAR reporter constructs.**
(TIFF)

Table S3 **Amino acid sequences encoding mutants of PAR1 reporter construct.**
(TIFF)
