# Supplementary figures and images for: Streptococcal SpeB Cleaved PAR-1 Suppresses ERK Phosphorylation and Blunts Thrombin-Induced Platelet Aggregation
Source: PLoS One. 2013 Nov 22;8(11):e81298. doi: 10.1371/journal.pone.0081298 (PMC3838405; doi:10.1371/journal.pone.0081298)

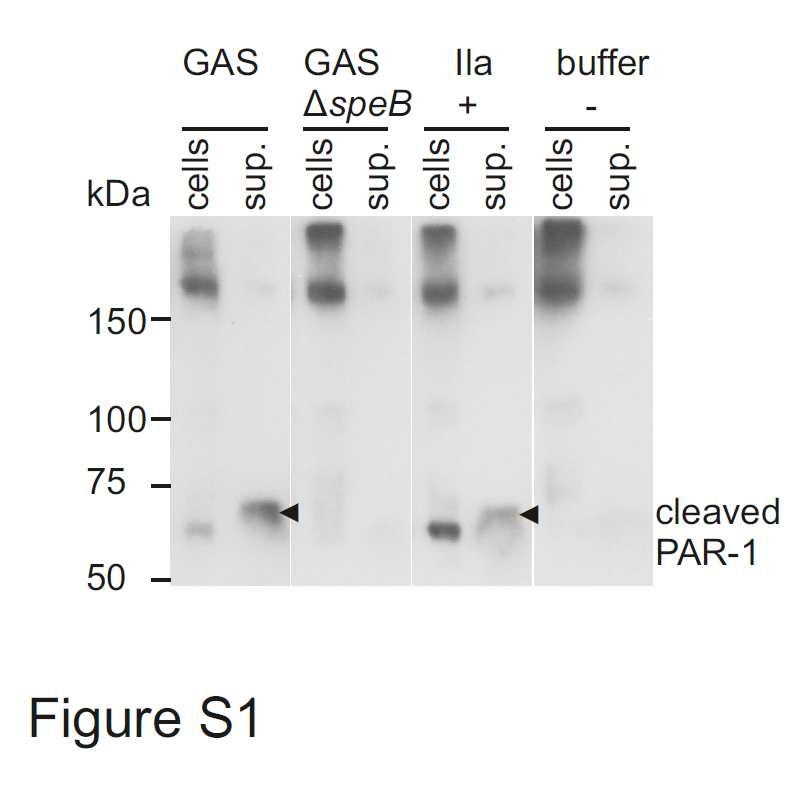

Supplement: Figure S1 — No cleavage of AP-PAR-1 construct outside PAR-1’s N-terminus. 293T cells transiently expressing alkaline phosphatase and FLAG-tagged PAR-1 were incubated with bacterial supernatants, thrombin (IIa; 1nM) or buffer. Supernatants and cells were then separately analysed by Western blot for N-terminal PAR-1 cleavage. Representative experiment out of 3. (TIF) [file pone.0081298.s001.tif]

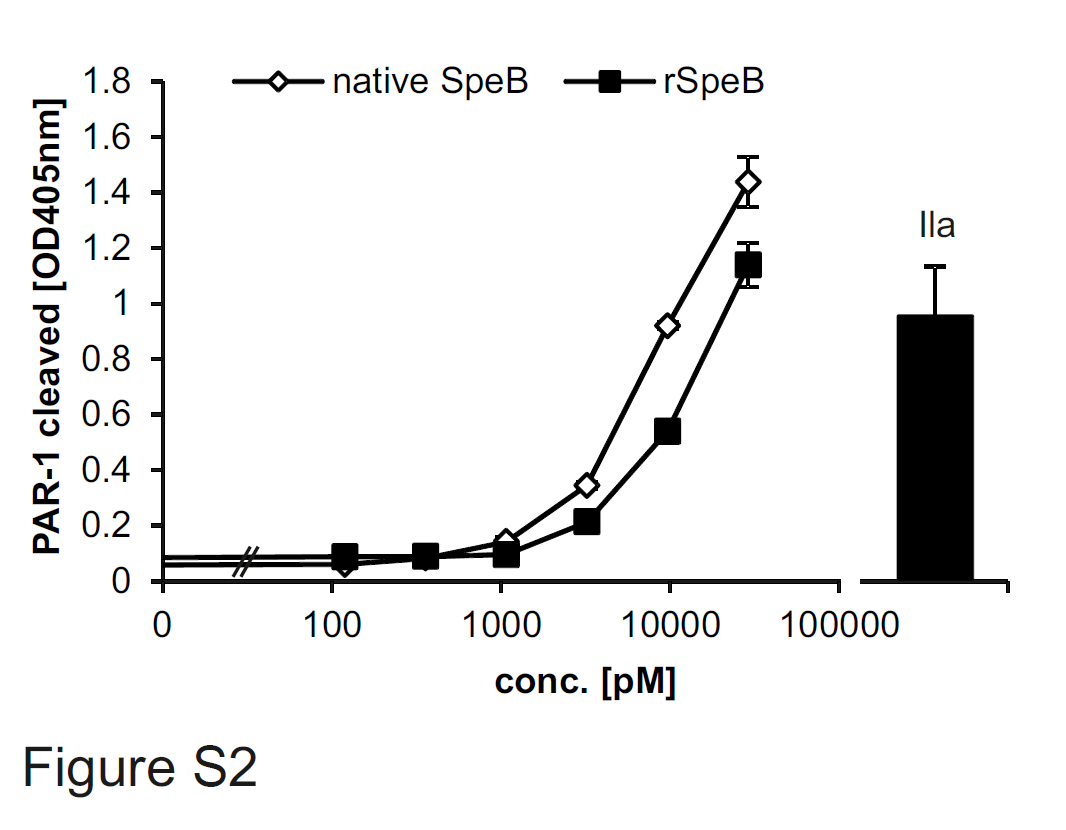

Supplement: Figure S2 — SpeB column purified from GAS supernatants and commercial SpeB cleaved PAR-1 with comparable efficiency. 293T cells transiently expressing alkaline phosphatase-tagged PAR-1 were incubated with indicated amounts of column purified (pSpeB) and commercial SpeB and cleavage of PAR-1 reporter constructs was quantified. Thrombin (IIa; 1nM) served as a positive control. (TIF) [file pone.0081298.s002.tif]
